# Supplementary material for: Defining Utility as a Measure of Preference Under Uncertainty in Phase I–II Oncology Dose Finding Trials
Source: Stat Med. 2026 Apr 30;45:e70547. doi: 10.1002/sim.70547 (PMC13130384; doi:10.1002/sim.70547)
Supplement: Supplementary file 1 — Data S1: Supporting Information. Figure C1. Example to visualize the effect of the interaction component of the joint utility function. All plots follow the utility independent relation in Equation (13) with simple risk neutral marginal utility functions, that is, uE=π and uT=1−π. For the Positive interaction plot, kE=0.25, kT=0.25 and kET=0.5. For the no interaction plot, kE=0.5, kT=0.5 and kET=0. For the negative interaction plot, kE=0.75, kT=0.75 and kET=−0.5. It can be seen how the slope of contour changes with utility for each of the different interactions. Figure E2. (A) Marginal efficacy utilities for the two utility functions in the simulation study, and (B) corresponding joint utilities, according to Equation (14), when toxicity probability is held fixed at selected values. Fixing one attribute linearly rescales the marginal utility of the other according to constants in the joint utility function and the marginal utility of the fixed attribute. Table E1. Listing of each of the probability and fixed trial parameters for simulation study. Figure E3. Plot of first six scenarios in simulation study Scenarios 1:6. Green line is the fixed probabilities for efficacy π˜E(D) and red line for toxicity π˜T(D). Dashed lines represent the cut points for the admissibility rules given in R2DT (1). Figure E4. Scenarios 7:10. Green line is the fixed probabilities for efficacy π˜E(D) and red line for toxicity π˜T(D). Dashed lines represent the cut points for the admissibility rules given in R2DT (1). Figure E5. Simulation Utility Functions: Contours in the joint utility represent equal utility at 0.1, 0.2, …, 0.9 with the point at guaranteed efficacy and no toxicity having utility of 1. Dashed lines are limits for admissibility rules. The contour plot for R2DT (3) and R2DT (4) gives the stopping rule (i) in black (u(0.5,0.35)=0.58), (ii) in red (u(0.7,0.4)=0.62) and (iii) in Green (u(0.9,0.4)=0.69). Table E2. Simulation study results applying novel stopping rule to EffToxU: [file SIM-45-0-s001.pdf]

A PROGRAMMING CODE

The R code to generate all data within the manuscript is provided on a public repository: https://github.com/medahala

B EFFTOX UTILITY DESIGN

The EffTox utility design[23,18] is used as a comparator to assess the *R2DT* in the simulation study. This section shows that the EffTox utility design can be formulated as a special case of *R2DT* that assumes simple risk neutral marginal utility functions.

The EffTox Utility design specifies a discrete utility function on the four possible individual patient level outcomes, $Y = (Y_E = a, Y_T = b)$, as follows

$$u(Y_E = a, Y_T = b) = \begin{cases} K(1,1), & \text{for } a = 1 \text{ and } b = 1 \\ K(0,0), & \text{for } a = 0 \text{ and } b = 0 \\ K(1,0), & \text{for } a = 1 \text{ and } b = 0 \\ K(0,1), & \text{for } a = 0 \text{ and } b = 1 \end{cases} \tag{B1}$$

Where $K(a,b)$ are constants to be specified. Given that utility is indifferent to linear transformations, $K(1,0) = 1$ and $K(0,1) = 0$ can be specified as the best and worst outcomes respectively. Expected utility is calculated by averaging the utility function over the chance of a state of nature (each patient outcome) happening. For the EffTox utility design the expectation is given by

$$E(u(Y(Y_E = a, Y_T = b)) = \int \sum_{a=0}^{1} \sum_{b=0}^{1} K(a,b)\pi_{a,b}, \tag{B2}$$

where π_{ab} represent the probability of an event happening. Assuming independence with $\pi_{11} = \pi_E\pi_T$, $\pi_{00} = (1 - \pi_E)(1 - \pi_T)$, $\pi_{10} = \pi_E(1-\pi_T)$, $\pi_{01} = (1-\pi_E)\pi_T$, and standardising with $K(0,1) = 0$ and $K(1,0) = 1$, the expectation equation can be rewritten as a function of π_E and π_T:

$$E(u(Y)) = E(u(\pi_E, \pi_T)) = \int_\theta K(1,1)\pi_E + K(0,0)(1 - \pi_T) + (1 - K(0,0) - K(1,1))\pi_E(1 - \pi_T)d\theta \tag{B3}$$

The expected utility equation can be written as a function of the population level parameters for the probability of an event at each dose. The specific equation has been written in this form as it is analogous to the utility independence equation, Equation 13 with $K(1,1) = k_E$, $K(0,0) = k_T$, $u_E = \pi_E$ and $u_T = 1 - \pi_T$. The marginal utility functions are the identity function or the degenerate case of R2DT, $\lambda_E = \lambda_T = \alpha_{GE} = \alpha_{LE} = \alpha_{GT} = \alpha_{LT} = 1$, with $\overline{\pi}_T$ and $\overline{\pi}_E$ becoming redundant in this special case due to the normalisation function. This demonstrates that the EffTox utility design can be formulated as a special case of R2DT that assumes simple risk neutral marginal utility functions with interpretation from the perspective of population level parameters. In the paper introduction it was stated the design was indifference to decisions under uncertainty as the marginal utility functions are linear (risk neutral).

C JOINT UTILITY

R2DT assumes a number of conditions to define the utility function in the form $u(\pi_E, \pi_T) = f(u_E(\pi_E), u_T(\pi_T))$ with $f(\cdot)$ a linear function, u_E a marginal utility function of π_E , and u_T a marginal utility function of π_T. These conditions and interpretation of additional parameters defined in $f(\cdot)$ are given in this section.

With attributes π_E, efficacy, and π_T toxicity, consider a point (e, t), within the domain of all possible levels $\pi_E \times \pi_T$, such that

$$0 \leq e \leq 1 \quad \text{and} \quad 0 \leq t \leq 1 \tag{C4}$$

Consider two conditional utility functions $u(e', \cdot)$ and $u(e'', \cdot)$ from two points e' and e''. Defining a lottery from the conditional utility function $u(e', \cdot)$ concerning two points t_1 and t_2 and associated certainty equivalent $\hat{t}$. We then contrast this with the certainty equivalent from the same lottery from the conditional utility function $u(e'', \cdot)$. If the certainty equivalent, $\hat{t}$ does not shift we can say that the two are strategically equivalent.

Efficacy is utility independent of toxicity when conditional preferences for lotteries on π_E given π_T do not depend on the particular level of t. When efficacy and toxicity are mutually utility independent we can express the utility function $u(e, t)$ in a multi-linear (bilinear) form as Equation 13,[37].

The marginal utility functions u_E and u_T do not depend on the level of the other attribute, as per the condition of mutual utility independence, as such these are referred to as efficacy and toxicity marginal utility functions for simplicity. The constant

k_{ET} represents an interaction between the two attributes. A smaller sum of k_E and k_T would constitute a greater interaction and $k_{ET} = 0$ no interaction.

When combining two measures of consequence through a function to give a single measure of consequence as is the case here it is necessary to have an understanding of what the function is achieving; the key to this is the interaction term.

The simplest case is the independent case, this is also called additive utility independence. With additive utility independence there is no interaction term ($k_{ET} = 0$) and the relationship between the two attributes is a simple linear payoff. There is only a single parameter that needs specifying since $k_T = 1 - k_E$. A small incremental increase in efficacy utility is directly proportional to a increase in toxicity utility (lower toxicity) with the magnitude of the constant dictating how much a small incremental increase in efficacy utility is worth in terms of the same increase toxicity utility. This simple payoff remains constant at all levels of efficacy and toxicity.

A positive interaction is when $k_E + k_T < 1$ and would imply that the higher the efficacy utility, the greater (more positive) the effect of toxicity utility (reduction in toxicity) on overall utility. Similarly, the higher the toxicity utility, the greater (more positive) the effect of efficacy utility on overall utility. The opposite being true of a negative value for the interaction parameter. This description of each possible interpretation for the interaction term is plotted in Figure C1. It can be seen for the plot with no interaction that the slope for toxicity with respect to efficacy is a constant at points within the joint domain. For a positive interaction the slope for toxicity with respect to efficacy is initially steep at the left hand end of the contour and reduces moving left to right. This suggests that as toxicity increases the effect of efficacy is reduced. The interpretation is synonymous with the clinical situation described for the motivating example in Section 1, with the effect of additional efficacy when there is high toxicity being minimal. A negative interaction describes the opposite to the situation in that the slope gets progressively steeper or the effect of additional efficacy becomes greater with more toxicity.

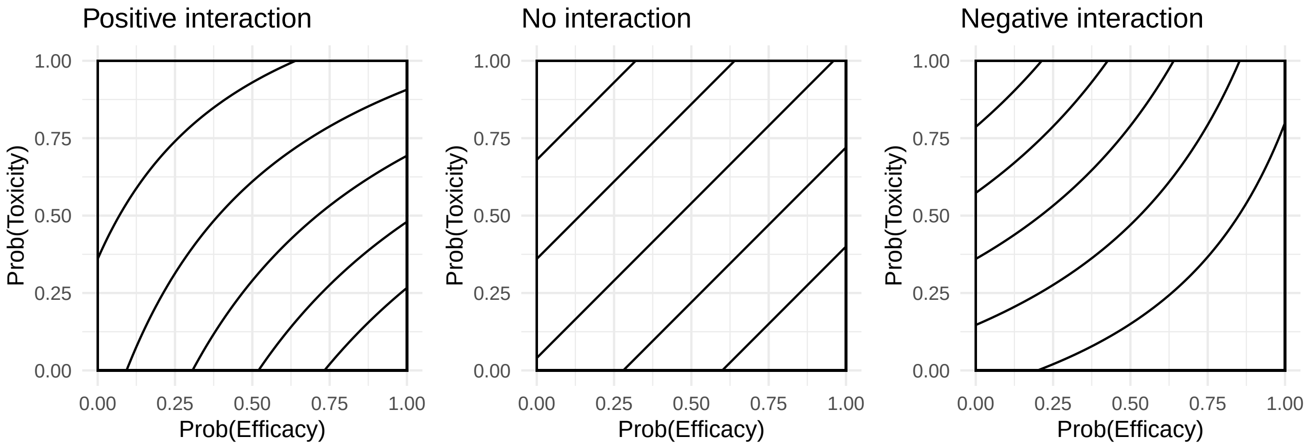

FIGURE C1 Example to visualise the effect of the interaction component of the joint utility function. All plots follow the utility independent relation in Equation 13 with simple risk neutral marginal utility functions i.e. $u_E = \pi$ and $u_T = 1 - \pi$. For the Positive interaction plot, $k_E = 0.25$, $k_T = 0.25$ and $k_{ET} = 0.5$. For the no interaction plot, $k_E = 0.5$, $k_T = 0.5$ and $k_{ET} = 0$. For the negative interaction plot, $k_E = 0.75$, $k_T = 0.75$ and $k_{ET} = -0.5$. It can be seen how the slope of contour changes with utility for each of the different interactions.

The Figure represents a simplification of the marginal utility functions. Considering the utility with positive interaction with respect to a reference dependence and whether each attribute is a gain or loss. The interpretation is that both attributes need to be a 'gain' for the overall utility to be considered likewise. In terms of losses, if one attribute is a loss this is almost as bad as if both attributes are losses - in both cases neither would likely be suitable to treat the wider population. This is the case in oncology dose finding settings where the payoff becomes more beneficial when both attributes improve.

D ADMISSIBLE CRITERIA

The admissibility criteria used as a comparator are defined separately in relation to cut points $\overline{\pi}_{addE}$ and $\overline{\pi}_{addT}$ and evidence levels p_E and p_T:

$$\Pr\{\pi_E < \overline{\pi}_{addE} \mid y\} > 1 - p_E \tag{D5}$$

$$\Pr\{\pi_T > \overline{\pi}_{addT} \mid y\} > 1 - p_T \tag{D6}$$

If either criteria is met the dose will be excluded from the set D. If all doses meet the criteria the trial is stopped.

E ADDITIONAL TABLES AND FIGURES

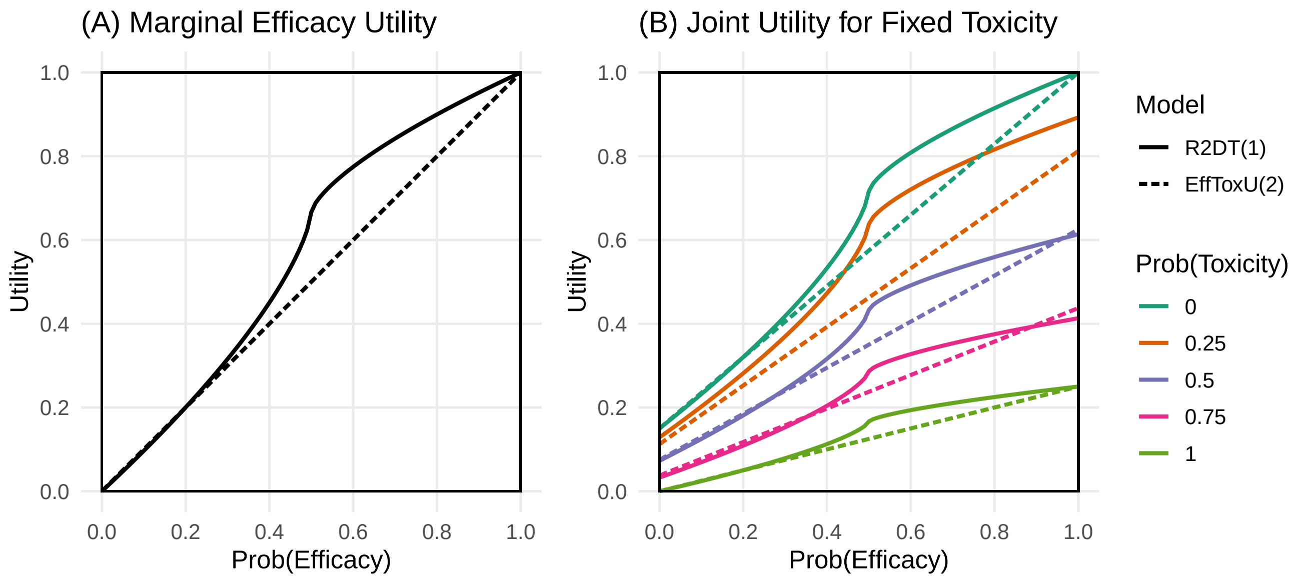

FIGURE E2 (A) Marginal efficacy utilities for the two utility functions in the simulation study, and (B) corresponding joint utilities, according to Equation 14, when toxicity probability is held fixed at selected values. Fixing one attribute linearly rescales the marginal utility of the other according to constants in the joint utility function and the marginal utility of the fixed attribute.

TABLE E1 Listing of each of the probability and fixed trial parameters for simulation study

Notation	Value	Interpretation
D	$[20, 30, 40, 50]$	actual doses
x	$[-0.5, -0.1, 0.19, 0.41]$	transformed doses
x^2	$[0.25, 0.01, 0.04, 0.17]$	square of transformed doses
α_T	$N(-3.17, 2.88)$	toxicity intercept
β_{1T}	$N(-3.56, 2.79)$	toxicity slope
α_E	$N(0.73, 2.44)$	efficacy intercept
β_{1E}	$N(-0.11, 2.34)$	efficacy slope
β_{2E}	$N(0, 0.2)$	efficacy squared slope
$\tilde{\pi}_E(D)$	$[0.42, 0.57, 0.67, 0.72]$	Efficacy prior probabilities
$\tilde{\pi}_T(D)$	$[0.14, 0.2, 0.26, 0.33]$	Toxicity prior probabilities
	$[1, 1]$	ESS toxicity and efficacy
	20	Starting dose
N	45	Max Sample Size
	3	Cohort Size
	2000	Number of simulation repetitions

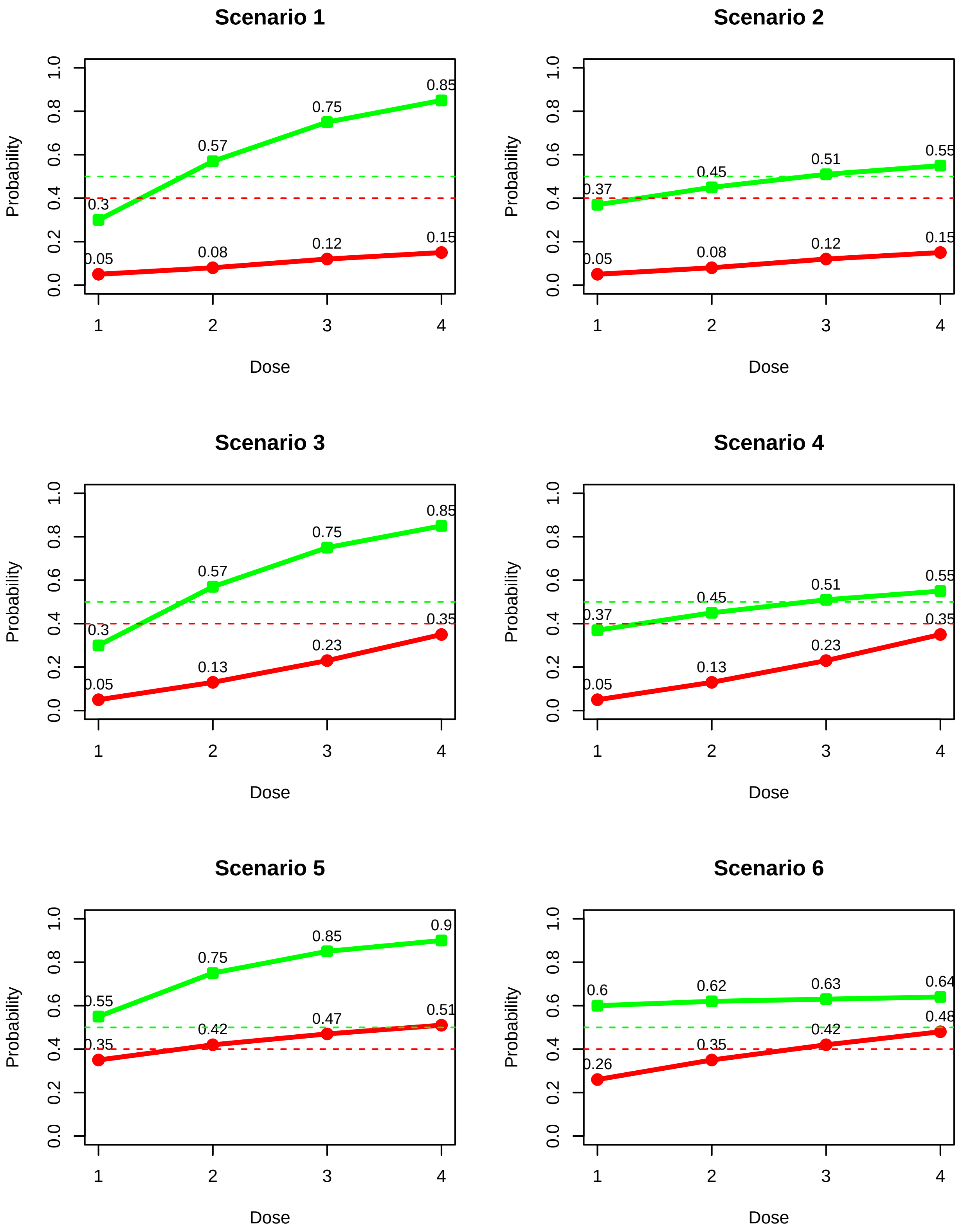

FIGURE E3 Plot of first six scenarios in simulation study Scenarios 1:6. Green line is the fixed probabilities for efficacy ($\tilde{\pi}_E(D)$) and red line for toxicity ($\tilde{\pi}_T(D)$). Dashed lines represent the cut points for the admissibility rules given in *R2DT (1)*

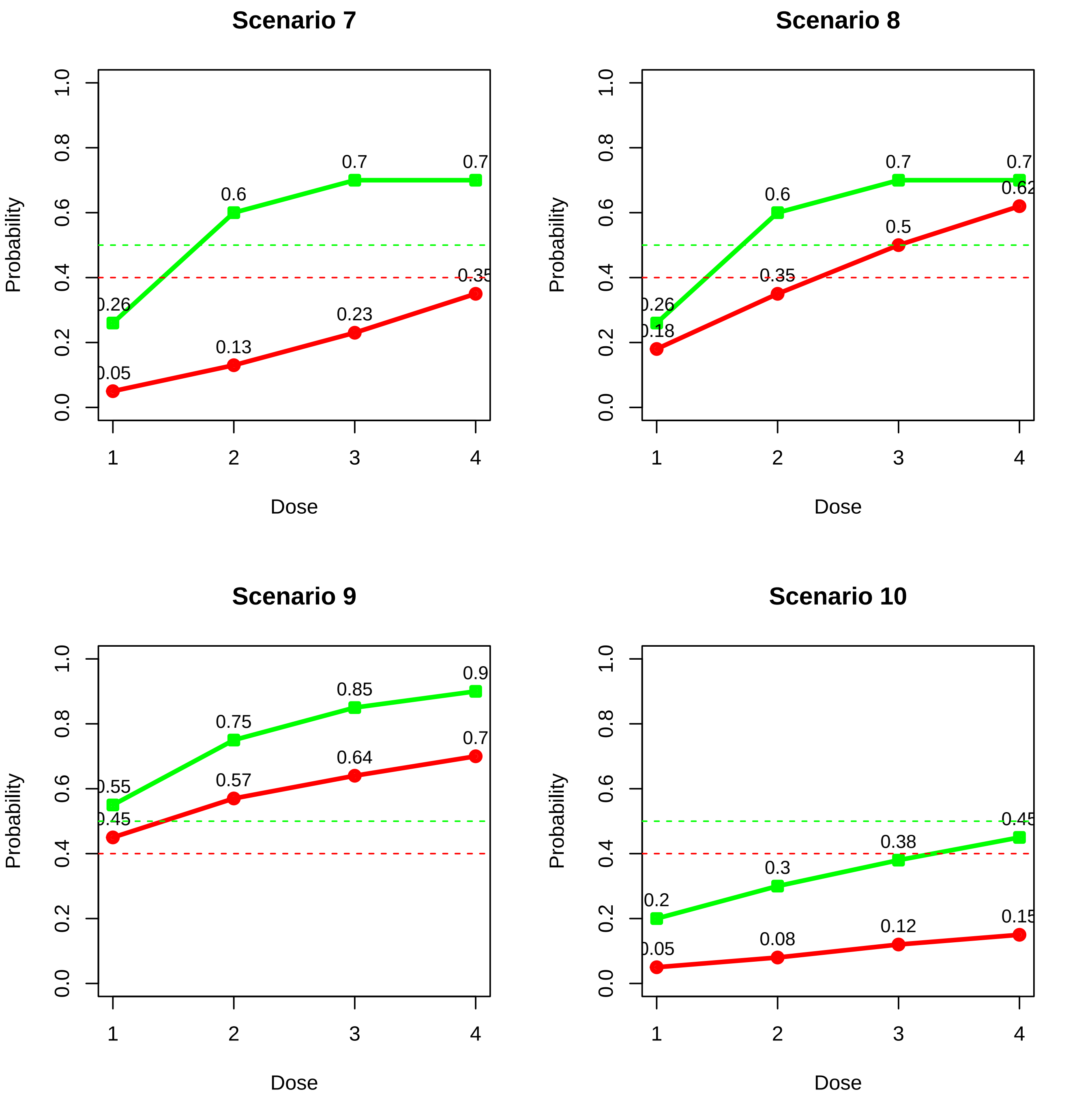

FIGURE E4 Scenarios 7:10. Green line is the fixed probabilities for efficacy ($\tilde{\pi}_E(D)$) and red line for toxicity ($\tilde{\pi}_T(D)$). Dashed lines represent the cut points for the admissibility rules given in *R2DT (1)*

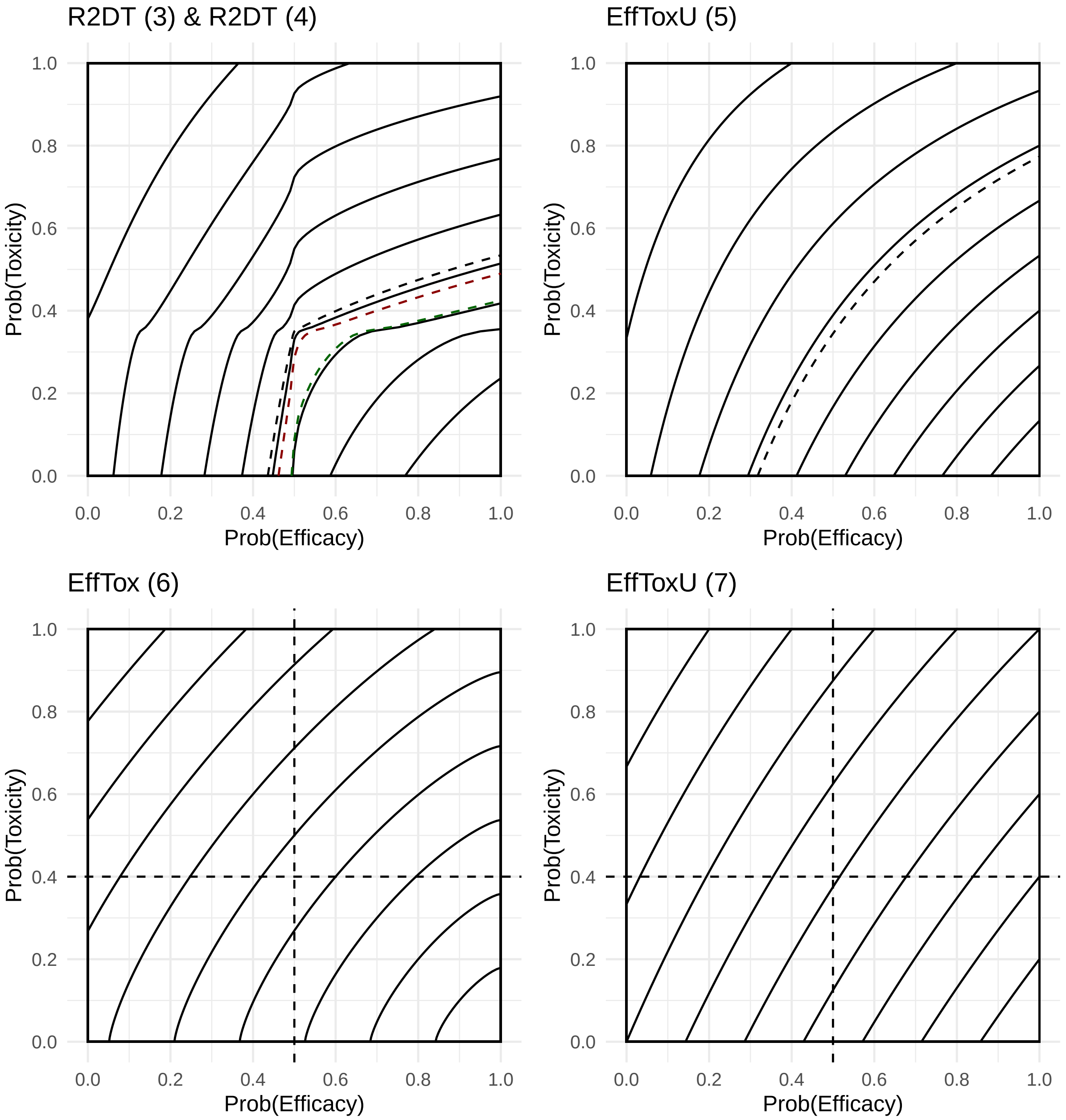

FIGURE E5 Simulation Utility Functions: Contours in the joint utility represent equal utility at 0.1,0.2,...,0.9 with the the point at guaranteed efficacy and no toxicity having utility of 1. Dashed lines are limits for admissibility rules. The contour plot for *R2DT (3)* and *R2DT (4)* gives the stopping rule *(i)* in black ($u(0.5, 0.35) = 0.58$), *(ii)* in red ($u(0.7, 0.4) = 0.62$) and *(iii* in Green ($u(0.9, 0.4) = 0.69$)

TABLE E2 Simulation study results applying novel stopping rule to *EffToxU*: data of form: [utility at scenario probability (π_E, π_T)] percentage selection (average number of patients treated). Percentage of trials with no dose selected abbreviated to NDS. Bold indicates optimal dose or whether trial should recommend not selecting a dose (and stop early)

	Dose (mg/kg)				
Method	20	30	40	50	NDS
	**Scenario 1** (π_E, π_T)				
	(0.3, 0.05)	(0.57, 0.08)	(0.75, 0.12)	(0.85, 0.15)	
EffToxU (2)	[0.39] 1.5 (4.8)	[0.60] 4.1 (5.3)	[0.72] 3.8 (4.1)	[**0.77**] 90.4 (30.8)	0.2
EffToxU (5)	[0.39] 3.2 (5.3)	[0.60] 4 (5.2)	[0.72] 3.6 (4.1)	[**0.77**] 89.1 (30.4)	0
	**Scenario 2** (π_E, π_T)				
	(0.37, 0.05)	(0.45, 0.08)	(0.51, 0.12)	(0.55, 0.15)	
EffToxU (2)	[0.45] 15.3 (11.6)	[0.50] 5.8 (5.9)	[0.53] 6.5 (4.2)	[**0.55**] 65 (21.8)	7.4
EffToxU (5)	[0.45] 24.9 (13.2)	[0.50] 6.2 (6)	[0.53] 5 (3.9)	[**0.55**] 63.5 (21.6)	0.5
	**Scenario 3** (π_E, π_T)				
	(0.3, 0.05)	(0.57, 0.13)	(0.75, 0.23)	(0.85, 0.35)	
EffToxU (2)	[0.39] 1.2 (4.8)	[0.57] 9 (6.7)	[**0.65**] 29.2 (10.2)	[0.64] 60.1 (23.1)	0.5
EffToxU (5)	[0.39] 2.9 (5.2)	[0.57] 8.8 (6.6)	[**0.65**] 28.3 (10.1)	[0.64] 60 (23)	0.1
	**Scenario 4** (π_E, π_T)				
	(0.37, 0.05)	(0.45, 0.13)	(0.51, 0.23)	(0.55, 0.35)	
EffToxU (2)	[0.45] 16.2 (11.8)	[0.48] 13.3 (7.5)	[**0.48**] 21.3 (7.4)	[0.45] 40.5 (16.4)	8.7
EffToxU (5)	[0.45] 28.2 (14.1)	[0.48] 13.5 (7.5)	[**0.48**] 17.6 (6.7)	[0.45] 38.4 (16)	2.2
	**Scenario 5** (π_E, π_T)				
	(0.55, 0.35)	(0.75, 0.42)	(0.85, 0.47)	(0.9, 0.51)	
EffToxU (2)	[0.45] 14.8 (7.6)	[0.54] 26.9 (11.4)	[0.56] 15.6 (7.3)	[**0.56**] 34.2 (17.2)	8.5
EffToxU (5)	[0.45] 8.1 (6.4)	[0.54] 20.6 (10.4)	[0.56] 16.6 (7.8)	[**0.56**] 54.6 (20.4)	0
	**Scenario 6** (π_E, π_T)				
	(0.6, 0.26)	(0.62, 0.35)	(0.63, 0.42)	(0.64, 0.48)	
EffToxU (2)	[**0.53**] 39.1 (14.8)	[0.49] 24.6 (12.8)	[0.46] 9.8 (5.8)	[0.44] 24.3 (11.2)	2.1
EffToxU (5)	[**0.53**] 38 (14.6)	[0.49] 22.8 (12.6)	[0.46] 8.6 (5.6)	[0.44] 29.8 (12)	0.8
	**Scenario 7** (π_E, π_T)				
	(0.26, 0.05)	(0.6, 0.13)	(0.7, 0.23)	(0.7, 0.35)	
EffToxU (2)	[0.36] 0.9 (4.6)	[0.59] 11.9 (7.4)	[**0.61**] 27.9 (9.4)	[0.54] 58.6 (23.4)	0.8
EffToxU (5)	[0.36] 2.2 (4.9)	[0.59] 11.8 (7.4)	[**0.61**] 27.1 (9.3)	[0.54] 58.6 (23.3)	0.4
	**Scenario 8** (π_E, π_T)				
	(0.26, 0.18)	(0.6, 0.35)	(0.7, 0.5)	(0.7, 0.62)	
EffToxU (2)	[0.32] 3.9 (6.3)	[**0.48**] 50.8 (14.4)	[0.46] 26.5 (10.6)	[0.39] 11.8 (12.3)	7
EffToxU (5)	[0.32] 5.9 (6.5)	[**0.48**] 28.3 (10.8)	[0.46] 24.1 (10.2)	[0.39] 35.7 (16.5)	5.9
	**Scenario 9** (π_E, π_T)				
	(0.55, 0.45)	(0.75, 0.57)	(0.85, 0.64)	(0.9, 0.7)	
EffToxU (2)	[0.40] 29.4 (12.3)	[0.45] 13.2 (8.9)	[0.45] 2 (4.3)	[0.43] 2.9 (8.6)	**52.5**
EffToxU (5)	[0.40] 13.7 (8.6)	[0.45] 20.8 (9.3)	[**0.45**] 13.4 (6.6)	[0.43] 48.9 (19.9)	3.2
	**Scenario 10** (π_E, π_T)				
	(0.2, 0.05)	(0.3, 0.08)	(0.38, 0.12)	(0.45, 0.15)	
EffToxU (2)	[0.31] 1.5 (6.1)	[0.38] 0.9 (3.7)	[0.43] 1.5 (3.6)	[0.47] 51.1 (21.7)	**44.9**
EffToxU (5)	[0.31] 6.1 (8)	[0.38] 1.9 (3.9)	[0.43] 2.4 (3.5)	[**0.47**] 77.8 (26.4)	11.8

TABLE E3 Sensitivity of EffToxU: data of form: [utility at scenario probability (π_E, π_T)] percentage selection (average number of patients treated). Percentage of trials with no dose selected abbreviated to NDS. Bold indicates optimal dose or whether trial should recommend not selecting a dose (and stop early)

Method	Dose (mg/kg)				NDS
	20	30	40	50	
**Scenario 1** (π_E, π_T)	(0.3, 0.05)	(0.57, 0.08)	(0.75, 0.12)	(0.85, 0.15)	
EffToxU (2)	[0.39] 1.5 (4.8)	[0.60] 4.1 (5.3)	[0.72] 3.8 (4.1)	[**0.77**] 90.4 (30.8)	0.2
EffTox (6)	[0.55] 1.4 (4.8)	[0.71] 4.2 (5.4)	[0.81] 3.5 (4.1)	[**0.85**] 90.6 (30.6)	0.2
EffToxU (7)	[0.49] 1.5 (4.8)	[0.67] 4.4 (5.3)	[0.77] 2.2 (3.7)	[**0.82**] 91.6 (31.2)	0.2
**Scenario 2** (π_E, π_T)	(0.37, 0.05)	(0.45, 0.08)	(0.51, 0.12)	(0.55, 0.15)	
EffToxU (2)	[0.45] 15.3 (11.6)	[0.50] 5.8 (5.9)	[0.53] 6.5 (4.2)	[**0.55**] 65 (21.8)	7.4
EffTox (6)	[0.59] 15.4 (11.7)	[0.64] 5.9 (6.1)	[0.66] 5.9 (4)	[**0.68**] 65.6 (21.8)	7.2
EffToxU (7)	[0.54] 15.3 (11.4)	[0.58] 6 (6)	[0.61] 6.6 (4.2)	[**0.62**] 65 (21.9)	7.1
**Scenario 3** (π_E, π_T)	(0.3, 0.05)	(0.57, 0.13)	(0.75, 0.23)	(0.85, 0.35)	
EffToxU (2)	[0.39] 1.2 (4.8)	[0.57] 9 (6.7)	[**0.65**] 29.2 (10.2)	[0.64] 60.1 (23.1)	0.5
EffTox (6)	[0.55] 1 (4.8)	[0.69] 8.4 (6.6)	[**0.76**] 31 (10.6)	[0.75] 59.2 (22.9)	0.4
EffToxU (7)	[0.49] 1.1 (4.8)	[0.64] 7.3 (6.2)	[0.72] 19.9 (8)	[**0.73**] 71.2 (25.9)	0.5
**Scenario 4** (π_E, π_T)	(0.37, 0.05)	(0.45, 0.13)	(0.51, 0.23)	(0.55, 0.35)	
EffToxU (2)	[0.45] 16.2 (11.8)	[0.48] 13.3 (7.5)	[**0.48**] 21.3 (7.4)	[0.45] 40.5 (16.4)	8.7
EffTox (6)	[0.59] 16.2 (11.8)	[**0.62**] 14.1 (7.7)	[0.62] 21.3 (7.5)	[0.60] 39.7 (16.1)	8.6
EffToxU (7)	[0.54] 17.2 (11.9)	[0.56] 13.2 (7.4)	[**0.56**] 18.4 (6.8)	[0.54] 43 (17)	8.2
**Scenario 5** (π_E, π_T)	(0.55, 0.35)	(0.75, 0.42)	(0.85, 0.47)	(0.9, 0.51)	
EffToxU (2)	[0.45] 14.8 (7.6)	[0.54] 26.9 (11.4)	[0.56] 15.6 (7.3)	[**0.56**] 34.2 (17.2)	8.5
EffTox (6)	[0.60] 14.1 (7.6)	[0.67] 27.7 (11.3)	[**0.69**] 15.3 (7.5)	[0.69] 34.4 (17.1)	8.5
EffToxU (7)	[0.54] 12.4 (7.2)	[0.64] 26.2 (10.7)	[0.67] 14.5 (7.1)	[**0.69**] 38.6 (18.6)	8.3
**Scenario 6** (π_E, π_T)	(0.6, 0.26)	(0.62, 0.35)	(0.63, 0.42)	(0.64, 0.48)	
EffToxU (2)	[**0.53**] 39.1 (14.8)	[0.49] 24.6 (12.8)	[0.46] 9.8 (5.8)	[0.44] 24.3 (11.2)	2.1
EffTox (6)	[**0.66**] 39.1 (14.8)	[0.63] 24.8 (12.8)	[0.61] 9.2 (5.8)	[0.58] 25 (11.3)	2
EffToxU (7)	[**0.61**] 40.6 (15.7)	[0.59] 21.4 (11.8)	[0.56] 9.7 (5.5)	[0.54] 26 (11.6)	2.1
**Scenario 7** (π_E, π_T)	(0.26, 0.05)	(0.6, 0.13)	(0.7, 0.23)	(0.7, 0.35)	
EffToxU (2)	[0.36] 0.9 (4.6)	[0.59] 11.9 (7.4)	[**0.61**] 27.9 (9.4)	[0.54] 58.6 (23.4)	0.8
EffTox (6)	[0.52] 0.9 (4.6)	[0.71] 12.7 (7.5)	[**0.73**] 27.4 (9.4)	[0.68] 58.4 (23.4)	0.7
EffToxU (7)	[0.46] 0.9 (4.7)	[0.66] 10.4 (6.8)	[**0.69**] 20.2 (7.8)	[0.64] 67.7 (25.5)	0.8
**Scenario 8** (π_E, π_T)	(0.26, 0.18)	(0.6, 0.35)	(0.7, 0.5)	(0.7, 0.62)	
EffToxU (2)	[0.32] 3.9 (6.3)	[**0.48**] 50.8 (14.4)	[0.46] 26.5 (10.6)	[0.39] 11.8 (12.3)	7
EffTox (6)	[0.49] 4.3 (6.6)	[**0.62**] 50.3 (14.4)	[0.60] 26 (10.4)	[0.54] 12 (12.4)	7.2
EffToxU (7)	[0.42] 4.7 (6.4)	[**0.57**] 42.8 (12.9)	[0.57] 30.8 (10.3)	[0.52] 14.6 (14.2)	7.2
**Scenario 9** (π_E, π_T)	(0.55, 0.45)	(0.75, 0.57)	(0.85, 0.64)	(0.9, 0.7)	
EffToxU (2)	[0.40] 29.4 (12.3)	[0.45] 13.2 (8.9)	[0.45] 2 (4.3)	[0.43] 2.9 (8.6)	**52.5**
EffTox (6)	[0.55] 30 (12.5)	[0.59] 13.2 (8.8)	[0.60] 1.6 (4.3)	[0.58] 3 (8.6)	**52.1**
EffToxU (7)	[0.50] 28.9 (11.9)	[0.57] 15 (9.1)	[0.59] 2 (4.5)	[0.59] 3.1 (8.8)	**51**
**Scenario 10** (π_E, π_T)	(0.2, 0.05)	(0.3, 0.08)	(0.38, 0.12)	(0.45, 0.15)	
EffToxU (2)	[0.31] 1.5 (6.1)	[0.38] 0.9 (3.7)	[0.43] 1.5 (3.6)	[0.47] 51.1 (21.7)	**44.9**
EffTox (6)	[0.49] 1.7 (6.1)	[0.54] 0.7 (3.7)	[0.58] 1.8 (3.6)	[0.61] 51.1 (21.7)	**44.8**
EffToxU (7)	[0.42] 1.4 (6.1)	[0.48] 0.8 (3.7)	[0.52] 1.8 (3.6)	[0.56] 51.2 (21.7)	**44.9**
